# Navigating the risks of prevention of mother to child transmission (PMTCT) of HIV services in Kibera, Kenya: Barriers to engaging and remaining in care

**DOI:** 10.1371/journal.pone.0191463

**Published:** 2018-01-24

**Authors:** Kerry A. Thomson, Barbara Telfer, Patricia Opondo Awiti, Jane Munge, Mathew Ngunga, Anthony Reid

**Affiliations:** 1 Médecins Sans Frontières (MSF) Operational Centre Brussels, Nairobi, Kenya; 2 Karolinska Institutet, Department of Public Health Sciences, Stockholm, Sweden; 3 Survey Research Team, Nairobi, Kenya; 4 Médecins Sans Frontières (MSF) Operational Centre Brussels, Luxembourg City, Luxembourg; Harvard Medical School, UNITED STATES

## Abstract

Within the first year of implementation, 43% of women who tested HIV positive at their first antenatal care visit were no longer retained and being followed in the free prevention of mother to child transmission (PMTCT) of HIV program offered by the Kenyan Ministry of Health and Médecins Sans Frontières in the informal settlement of Kibera, Nairobi. This study aimed to explore barriers to enrolling and remaining engaged in PMTCT services throughout the pregnancy and postpartum periods. Qualitative data from 31 focus group discussions and 35 in-depth interviews across six stakeholder groups that included women, men, and PMTCT service providers were analyzed. Using an inductive exploratory approach, four researchers coded the data and identified key themes. Five themes emerged from the data that may influence attrition from PMTCT service in this setting: 1) HIV in the context of Kibera, 2) knowledge of HIV status, 3) knowledge of PMTCT, 4) disclosure of HIV status, and 5) male partner support for PMTCT services. A new HIV diagnosis during pregnancy immediately triggered an ongoing risk assessment of perceived hazards in the home, community, and clinic environments that could occur as a result of female participation in PMTCT services. Male partners were a major influence in this risk assessment, but were generally unaware of PMTCT services. To preserve relationships with male partners, meet community expectations of womanhood, and maintain confidentiality while following recommendations of healthcare providers, women had to continuously weigh the risks and benefits of PMTCT services and interventions. Community-based HIV testing and PMTCT education, male involvement in antenatal care, and counseling customized to assist each woman in her own unique risk assessment, may improve uptake of and retention in care and optimize the HIV prevention benefit of PMTCT interventions.

## Introduction

Despite the availability of evidence-based interventions to prevent mother to child transmission (PMTCT) of HIV, an estimated 150,000 incident HIV infections and 110,000 AIDS related deaths occurred among children less than 15 years of age in 2015 [1]. In 2011, global and national leaders set ambitious targets as part of The Global Plan to substantially decrease new HIV infections in children and reduce mortality among mothers living with HIV, including a 90% reduction in child HIV infections, a 50% reduction in AIDS-related maternal deaths, and virtual elimination of mother to child transmission (eMTCT: defined as reducing mother to child transmission (MTCT) to less than 5%) [2, 3]. Furthermore, in 2014, the Joint United Nations Programme on HIV/AIDS (UNAIDS) set new targets intended to end the HIV epidemic, including having 90% of HIV positive individuals on antiretroviral therapy (ART) and sustaining viral suppression. Engaging and retaining women of reproductive age in HIV care, especially pregnant women, is central to achieving these ambitious targets. The Global Plan identified Kenya as one of 22 high priority countries for improved PMTCT services [4]; in this country with a high HIV burden, infants and young children less than 15 years of age account for 16% of all new HIV infections, mainly as a result of MTCT [5].

Pregnancy presents a window of opportunity to identify HIV infection and engage women and their partners in HIV treatment for their own health, and the health of their future children. To achieve eMTCT, HIV-positive women and their babies must be enrolled and retained in PMTCT programs and adhere to ART. However, loss to follow-up (LTFU), when women disengage from clinic-based services completely and have unknown pregnancy outcomes, is a significant challenge for programs throughout the PMTCT cascade of services in resource-limited settings. Recent review articles estimate that maternal adherence to antiretrovirals and LTFU of mother-infant pairs from PMTCT programs in Sub-Saharan Africa (SSA) range from 35–86% [6] and 19%-90%, respectively [7].

Prior studies on attrition from PMTCT programs in African settings have primarily gathered data from HIV-positive women only, and identified discrete factors that influence their participation in PMTCT services. Commonly cited barriers include stigma associated with HIV [6–9], limited understanding of PMTCT [8–10], fear of disclosure and lack of support from male partners [6, 7, 9, 10], and dissatisfaction with clinic programs, staff, and confidentiality [6, 8, 10]. There has been less in-depth analysis on the complex relationships between these barriers, and how external influences, including the community, male partners, and healthcare workers, contribute toward a woman’s decisions regarding enrollment and participation in PMTCT services.

In the first year (2006–2007) of the Médecins Sans Frontières (MSF) PMTCT program in Kibera, Kenya HIV prevalence was 13% among 2,262 pregnant women tested at initiation of routine antenatal care (ANC) [11]. Only sixty five percent of the 305 women who first tested HIV positive at ANC elected to enroll in PMTCT, and only half of these women remained in care until delivery [11]. We conducted the current study to explore factors that influence women’s experiences with HIV treatment and prevention in Kibera, with a specific focus on PMTCT services for HIV infected women. To our knowledge, this is the first qualitative study on PMTCT participation that focuses on the unique environment of the informal settlement of Kibera, and comprehensively draws on the different perspectives of male, female, and service provider stakeholder groups.

## Methods

### Study area

Kibera is a large, informal settlement located on the outskirts of Nairobi, Kenya. Although population estimates for Kibera have fluctuated from 300,000 to one million inhabitants [12, 13], with a total size of only 2.5 square kilometers the area is densely populated. Many Kibera residents migrate from rural areas in search of economic opportunities in Nairobi, and then live in crowded and impoverished conditions. Dwellings are small, semi-permanent structures made of mud, wooden planks, or metal sheets. Infrastructure for clean water, sanitation, and waste management is very limited.

### Médecins Sans Frontières services in Kibera

In partnership with the Ministry of Health, MSF has provided HIV/AIDS, tuberculosis, and primary health care services since 2003 at three clinics in Kibera, averaging 1,000 HIV patient visits per month across the three clinics [14]. Starting at its inception in 2006 and during the course of the current study, the MSF PMTCT program followed the 2006 WHO guidelines, including initiation of therapeutic highly active antiretroviral therapy (HAART) or short-course dual therapy (depending on maternal CD4 cell count and WHO staging), safe delivery, infant prophylaxis, and replacement feeding (when acceptable, feasible, affordable, sustainable, and safe) or exclusive breastfeeding for six months [15, 16]. All PMTCT services offered, including ART, facility delivery, and supplies for safe replacement feeding were provided free of charge. Women were offered enrollment in the PMTCT program after testing HIV-positive at their first ANC visit, by referral from the cohort of HIV-positive women already in care with MSF who became pregnant, or by testing HIV-positive at 34–36 weeks gestation when pregnant women were re-tested. PMTCT services were integrated into routine ANC services at two of the largest MSF clinics in Kibera; all pregnant women were seen in the same clinic environment regardless of HIV status. All women, and especially those who tested HIV positive and enrolled in the PMTCT program, were encouraged to bring their male partner to the clinic for HIV testing, counseling, and education.

### Study design

Qualitative methods were selected for the current study because it was exploratory in nature. We followed this exploratory and inductive approach throughout both data collection and analysis, allowing factors that influence engagement with PMTCT within the Kibera environment to emerge from the data. The research team did not seek to evaluate a pre-specified theory, however it was hypothesized that individuals external to pregnant women could have an influence on health-seeking behaviors. To ensure we captured this broad range of influence, we intentionally collected data across a range of six stakeholder groups, each of whom offered a unique perspective on the context of health, HIV treatment, and prevention in Kibera.

### Data collection

In-depth interviews (IDI) and focus group discussions (FGD) were conducted with six stakeholder groups, including women, men, and PMTCT service providers, between September 11 and October 26, 2008. An overview of data collection procedures, including the rationale for each stakeholder group is presented in Table 1. Participants were recruited using non-random purposive sampling [17]. Six semi-structured interview guides were created and customized for each stakeholder group. Each interview guide was pre-tested with a member of the respective stakeholder group who did not participate in the final data collection. The pre-test process solicited feedback on the appropriateness and scope of questions as well as use of clinical language and terminology, and informed revisions to the interview guides prior to the initiation of data collection. FGDs and IDIs were conducted by a trained moderator in either English or Kiswahili, based on the preference of the participant(s). All study participants were 18 years of age or older and gave verbal consent to participate. FGDs and IDIs were audio-recorded. A note taker was present during each FGD and IDI to record relevant observations. FGDs and IDIs with each stakeholder group were conducted in parallel and one researcher listened to the tapes and reviewed notes from each FGD and IDI during the data collection phase. Concurrent review of the data informed additional modifications to the interview guides, as needed. Through this iterative approach to data collection, researchers were able to assess when saturation had been reached (i.e. no new insights emerging from the data) [18–20]. Saturation was reached and data collection was complete after 31 FGDs and 35 IDIs were conducted.

**Table 1:** Overview of data collection methods for PMTCT qualitative study in Kibera, Kenya.

Stakeholder Group	Unique Perspective and Question Topics	HIV Status	Sampling Approach	Inclusion Criteria, Recruitment, Setting	Total Number of Interviews	Mean Number (Range) Participants per Group
**Focus Group Discussions (FGD)**						
Women at 1^st^ Antenatal Care Visit (ANC)	Women’s knowledge and attitudes on HIV and PMTCT prior to knowing their HIV status.	Unknown (prior to testing)	Convenience	Pregnant women were recruited at their first ANC visit. For women who consented to enroll in the study, FGD questions were added to the beginning of the standard group health education session conducted prior to HIV testing. Patients who declined study participation received the standard group health education session.	9	6 (5–7)
HIV positive women with at least 3 ANC/PMTCT visits	Factors that facilitated participation in PMTCT services.	HIV positive	Purposive	HIV positive women who completed a minimum of three ANC/PMTCT visits during pregnancy. Conducted at clinic or local maternity hospital.	9	6 (5–6)
Men Residing in Kibera	Men’s knowledge and attitudes toward HIV and PMTCT, and the influence male partners could have on PMTCT participation.	Unknown	Convenience	Men ages 18–49, any marital status and unknown HIV status, living in community surrounding the clinic. Conducted at a community church.	13	6 (5–9)
**In-depth Interviews**						
Women with ≥ 1 missed appointments for PMTCT	Reasons for missed appointments, or complete disengagement from the PMTCT program.	HIV positive	Purposive	HIV positive women who enrolled in PMTCT program, but missed one or more appointments, were contacted via phone or in-person by a clinic social worker. Conducted at a local maternity hospital. Women were invited to resume participating in HIV care, if appropriate.	13	1 per interview
Supportive Male Partners	Attitudes of male partners who were aware of their female partners HIV positive status and chose to be supportive of PMTCT services	Male status unknown, female partner HIV positive	Purposive	Men who came to PMTCT appointments with their HIV positive partners. Conducted at home or clinic.	9	1 per interview
PMTCT Service Providers	Information gleaned from clinicians providing PMTCT services.	Unknown	Purposive	Clinicians who worked in the PMTCT / ANC program. Conducted at the MSF clinic.	13	1 per interview

### Data analysis

Tapes were transcribed verbatim and translated into English. Data analysis was conducted by four qualitative researchers and followed an exploratory approach based on grounded theory [21, 22]. We used OpenCode software for data management and coding [23]. First, each member of the analysis team read through each transcript independently and identified possible inductive codes that emerged from the data. Next, the analysis team defined and agreed on a preliminary codebook of 30 codes. A pilot coding exercise was conducted where each researcher completed line-by-line open coding of selected transcripts. One researcher compared the results of the pilot coding exercise and facilitated a discussion to agree on a consistent coding approach. Then, each researcher manually coded every other transcript for each stakeholder group and captured observations in analysis memos. Analysts met routinely to discuss individual observations and reach consensus on new codes. After coding, each researcher reviewed the transcripts again by reading the text *down* and *across* each stakeholder group. Through this process of content analysis, the data from the different stakeholders were triangulated and condensed. Coding and preliminary analysis continued until no new themes emerged from the data. Codes were grouped into categories, and then categories were formed into five themes. Quotes were selected from the coded transcripts to illustrate these themes.

### Ethics

Study participants were 18 years of age or older and gave voluntary informed consent to participate. During a feasibility assessment conducted prior to data collection, community members, MSF patients, and staff explained they were unwilling to provide full names and a written signature or thumbprint on research documents for an HIV study; such signatures were restricted to official business with financial and government institutions. Thus, the final protocol obtained verbal informed consent only. A consent form specific to each stakeholder group was read aloud and verbal informed consent was obtained from each participant prior to data collection. The consent process was documented by the note taker who listed the first name of participant, place, and date of consenting. The informed consent process emphasized the potential risk of HIV disclosure and that participation had no effect on an individual’s ability to receive medical care at the MSF clinics. Women who had tested HIV-positive when initiating ANC and subsequently completed at least 3 ANC/PMTCT visits were recruited individually and informed that participation in the group would implicitly reveal their HIV status to other participants in the FGD only. Both FGDs and IDIs were conducted in private locations, and all FGD participants were asked to respect the confidentiality of others by not repeating anything said during the discussion. The study protocol, including informed consent procedures, was approved by the Kenyatta National Hospital Ethical Research Committee in Nairobi, Kenya and the Ethics Review Board of Médecins Sans Frontières.

## Results

**Fig 1:**
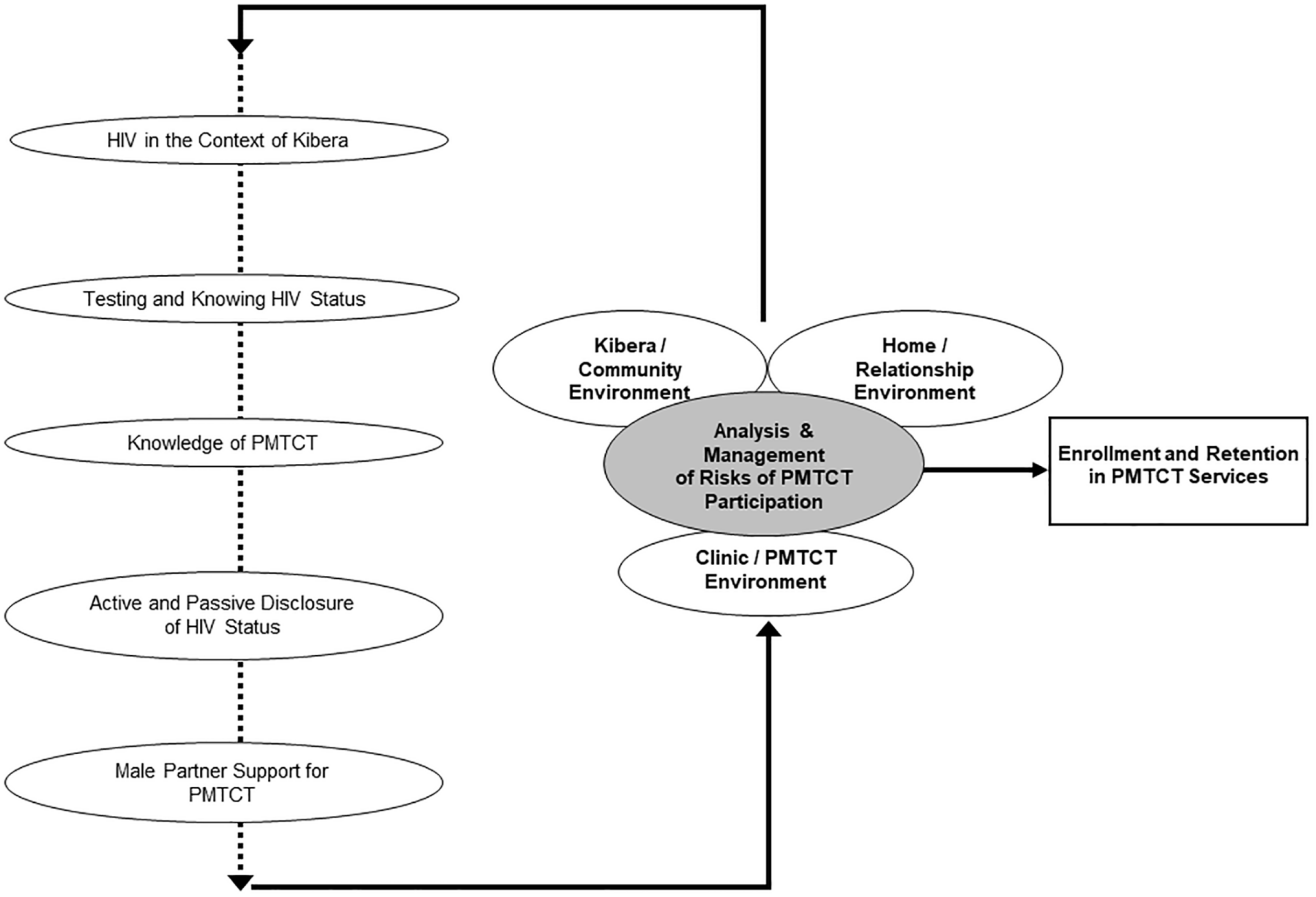
Summary of themes influencing enrollment and retention of HIV infected pregnant women in PMTCT services in Kibera, Kenya.

A total of five themes were identified from the data for why women do not fully engage in PMTCT services (Fig 1).

### 1. HIV in the context of Kibera

A number of physical and socioeconomic aspects of the environment of Kibera were not supportive of health-promoting behaviors, including conditions unfavorable to HIV prevention and seeking HIV testing. Substance abuse was common among men in light of the high unemployment rate and availability of the local brew at low prices. Commercial sex was available, and men were more likely to engage in high risk behavior while consuming alcohol. In addition to commercial sex workers, other women were forced by circumstances to exchange sex for material goods, food and/or physical security.

*… lack of resources and poverty is a major contributor [to HIV]. There are so many ladies without husbands and in the process of looking for food and livelihood they indulge in drinking and end up engaging in immoral activities out of being drunk*.

--Man Residing in Kibera

*They [men] know it [HIV] is there, but after they get drunk, they forget it is infectious*.

--Woman at 1^st^ ANC Visit

Knowledge of HIV transmission and prevention varied widely among study participants. Unprotected sex, contaminated sharp objects such as razors, and blood were frequently cited as routes of transmission. However, cultural misperceptions about HIV transmission still existed in Kibera, particularly among people who had come from rural areas. Study participants reported that some people still believed in “chiraa”, the idea that HIV was caused by someone putting a spell on the infected person, or punishment for having inappropriate sexual relationships. Using condoms, monogamy, and abstinence were acknowledged as means to prevent HIV transmission, but men seemed reluctant and women were not empowered to actually implement these prevention strategies in their everyday lives.

*Men can decide whether to be infected or not, but women cannot. A man can force a woman to have sex without a condom, but a woman cannot. So, men have the upper hand of being protected against HIV, but women are at [the] mercy of men*.

--Man Residing in Kibera

Men reported that they did not want to use condoms during sex, especially with their wives. In turn, women reported being powerless to negotiate condom use. Men, and to a lesser extent women, reported having multiple partners, and sexual relationships outside of established partnerships were frequently disclosed.

*These men are not faithful and that is why they stop the women from getting tested or going for the services or giving them support. And most likely he knows that he is [HIV] positive from having other relationships so he does not want the woman to get tested. Maybe even the woman wants to go but the man stops her saying she is just okay and does not need the services. This man does not want his deeds to be discovered*.

--Man Residing in Kibera

Both men and women were aware that HIV testing and treatment were available, and provided free of charge at the clinic. Despite this, many study participants viewed HIV as a fatal condition, and seemed unaware that it could be effectively managed as a chronic disease. While some respondents had a positive attitude towards care and treatment for people living with HIV, others reported that ART was a problem since it kept infected people alive and well, further facilitating transmission of HIV.

*[Not seeking services] is related to fear of being seen there. Someone will go and spread word that they have seen you at the HIV place and believe you have HIV. So, this will make someone come once and the next time they hide*.

*The best thing they should do is to stop giving people with HIV drugs, they should be left to die… Because if it is not stopped, they will continue getting healthy, fat, and beautiful*.

--Men Residing in Kibera

In Kibera, the stressors of securing work, food, and housing created an environment of constant insecurity for men and women. In the face of this daily struggle it was difficult for many Kibera residents to prioritize matters of preventive health and treatment, including HIV.

I have five children, the one I am carrying is my sixth child so the milk [formula] I get, I swear before God I sell it so that I can get money to buy flour for the other kids to also eat. Because this child cannot feed alone while the others go hungry. I am making a mistake but what else can I do?

--Woman with at Least 3 ANC Visits

### 2. Testing and knowing HIV status

Stigma and fear of HIV was common among residents in Kibera. Although testing sites were available, actively seeking out voluntary HIV counseling and testing (VCT) was not the community norm, particularly among men. During FGDs, men and women acknowledged the importance of knowing one’s status in the abstract, but reported that community members were reluctant to go and get tested. Denial, complacency, fear of death, and the anticipation of unbearable stress were cited as reasons why people avoided HIV testing. People were more likely to seek out testing if they felt ill, had had a partner die, or learned that their partner was HIV-positive. Men in particular were unlikely to undergo a HIV test or to seek health care, unless gravely ill. Generally, women and most men were aware that HIV testing was a routine part of ANC services. Therefore, women were most often the first partner within the relationship to be tested. In this context whoever learned of their HIV status first was viewed as the partner responsible for acquiring the virus first.

*If she is [HIV] positive and I am [HIV] negative then she must have been playing some bad games outside. So she will go. Because I have been faithful to her and yet she has become a murderer*.

*I have also seen that fear in another couple. The woman was pregnant and wanted to go to the clinic [with HIV services] but the man refused and told her to go to a midwife. The woman used other ways and went to the clinic and got tested [for HIV] and when the man got to know about it, she was beaten. So this can also cause fear*.

--Men Residing in Kibera

When a woman tested HIV-positive at first ANC and disclosed to her partner, it prompted some men to actively seek out testing for themselves; other men just assumed that they would be also positive. Male and female study participants expressed very different reactions to HIV diagnosis. After the initial shock, women reacted with more acceptance than men. Women seemed to approach HIV as yet another challenge to deal with in their already hectic lives and were more open to managing HIV as a chronic disease.

*A man is the head of the house, they see [HIV diagnosis] as if they are already dead and they have left behind their family and dependents. A woman sees it as any other disease which can be managed*.

--Man Residing in Kibera

### 3. Knowledge of PMTCT

Awareness and knowledge specific to PMTCT services was limited among study participants in all lay stakeholder groups. Participants reported that sexual intercourse during pregnancy, delivery (especially cutting of the umbilical cord), and breastfeeding were opportunities for vertical transmission and some male and female participants could describe one or two discrete PMTCT interventions (e.g. facility delivery, taking medication, replacement feeding). There was limited understanding of PMTCT as an integrated package of prevention services and interventions spread over a period of time throughout the pregnancy and the postpartum period, and how these PMTCT services could prevent a baby from being infected with HIV.

Female patients in all stakeholder groups expressed that most information and education about prevention of vertical HIV transmission is directed at women only, but that women may not be able to freely share what they have learned with male partners. Because men rarely visited health clinics themselves, they were largely dependent on female partners as the primary source of information on PMTCT services. Some respondents felt that information on the prevention benefits of PMTCT services was only available within the clinic, and mainly for HIV-positive people, rather than for the Kibera community at large.

*The outsiders [community members] do not know about PMTCT, only those who come for the services know about it*.

--Woman with ≥ 1 missed appointment for PMTCT

Even amongst themselves, women did not discuss PMTCT openly or serve as a resource to other women.

*Many don't talk about these [PMTCT services] because when you start dwelling on the topic people suspect you, and someone would not want to be known if they have the [HIV] virus*.

--Woman at 1^st^ ANC

### 4. Disclosure of HIV status

Disclosing HIV status to a male partner was the most complicated disclosure event and generated the most discussion. To a lesser extent, women disclosed to family members, primarily their mothers or a sibling. Women had little trust in female friends or neighbors and rarely described disclosing to them. An exception was seen among women who had attended three or more PMTCT visits. Some of these women were fully open about their HIV status in the community, and had tried to be a resource for other HIV-positive women. However, study participants perceived that, overall, there was a lack of support for people living with HIV within the Kibera community. Stigma persisted as a key barrier to disclosure. Both the FGDs and interviews revealed that disclosure of HIV status was a complex and multi-faceted issue for men and women. Our results revealed that disclosing HIV status to others can be conceptualized as either an active or passive process.

#### Active disclosure

Active disclosure was described as an explicit action, most commonly a verbal conversation with the intention of sharing one’s HIV status. Because a woman typically was the first member of a couple to be tested (at initiation of ANC) the onus was on her to disclose to her male partner and initiate a conversation about HIV and the benefits of participating in the PMTCT program. This expectation for disclosure came during pregnancy, a time when women were often physically and economically vulnerable and even more dependent on their partner for survival. In some situations, a male partner had already been tested and knew his HIV status, but men were not subject to the same pressures to disclose to their female partner.

*I would support her [pregnant partner] to go for the test. If she is found [HIV] positive, that would be the end of my support*.

--Man Residing in Kibera

Women described how they weighed the pros and cons of disclosing to their male partners and tried to assess their partner’s reaction in advance. Fear of physical violence, rejection, or being abandoned prevented many women from disclosing to their partner.

*You know sometimes you can be [HIV] positive and the husband is negative and as a result you are kicked out, so to avoid this you just keep quiet*.

--Woman at 1^st^ ANC Visit

Women also feared accusations of infidelity and being blamed for transmission of HIV especially in serodiscordant couples.

*He will beat you badly. He'll say that it is you who has brought the [HIV] disease home, but it was actually himself*.

--Woman at 1^st^ ANC Visit

Female participants who had disclosed their positive HIV status described a range of reactions. Some men did react with denial, violence, and abandoning their partner. Thus, for many women, active disclosure could end their relationship and break up their household.

*I did not know my [HIV] status [until ANC] and now that he knew I was pregnant he told me he doesn’t want [baby]. He went alone and got tested. He was found [HIV] negative and that's when he ran away from me. He told me he doesn't want a baby in the house he did not leave money for food. I was living here and when he left me I was forced to go stay at my mother's. Nowadays I come from my mother’s to the clinic because at home you can't get milk [infant formula] it's expensive*.

--Woman with ≥ 1 missed appointment for PMTCT

Some men residing in Kibera confirmed this, asserting that if their spouse went for an HIV test and was positive, she would have to leave the household immediately.

*Most men do chase away their spouse or go separate ways once they learn of such things [HIV positive female partner]. That is the reality on the ground. Most homes are broken due to such like things*.

--Man Residing in Kibera

Other men, supportive male partners, responded by going for an HIV test, and gradually accepted their HIV-positive diagnosis.

*My wife was the first to come for VCT and it was found she in [HIV] infected. She shared it with me. Thereafter we sat together and discussed about the issue and we saw it is true and we couldn't leave each other because we were both infected and we decided to stay together and started using drugs*.

--Supportive Male Partner

A women’s decision to disclose was also dependent on the strength of the existing relationship. Based on shared experiences as a couple, their level of communication, and his reaction to prior stressful circumstances, a woman assessed whether disclosing would be safe and facilitate participation in PMTCT services.

*I am a man and I cannot have only one wife, I must have another one as a side plate. I will not allow my wife to attend PMTCT clinic because that will make her know my secret. If she is tested and she is positive, automatically I will know that I am also infected. And as you know, we men we are always tough in arguments. She will not come to me and say, 'Daddy, I went there and got tested and I am positive'. That is the time I will send her away from my home*.

--Man Residing in Kibera

#### Passive disclosure

Passive disclosure was described as circumstances that inadvertently exposed someone’s HIV-positive status to others. Although they offered a full spectrum of primary care, the MSF clinics in Kibera were predominantly perceived as HIV clinics. As such, both men and women felt their HIV-positive status was being disclosed by attending the clinics. Even though PMTCT services were integrated into routine ANC and there was not a separate “HIV Clinic”, they feared being seen and recognized while attending clinic appointments. This was a concern for women at their first ANC visit, and continued to be a recurring fear during subsequent visits. Long waiting times at the clinics increased the risk of being seen.

*When a mother turns out [HIV] positive, she fears being blamed by the husband that she may be the one who brought in the virus, so she keeps to herself and refuses to go to the clinic [again] lest her husband gets to know the truth about her status because she may be given drugs and the husband may see them and question her about them. Some women fear being tested, they know that when a pregnant woman comes to the clinic, it is a must for her to be tested for HIV. So they fear the outcome, they keep away from the clinic. Some women also fear being seen by the people who know them coming to the clinic*.

--Service Provider

Some PMTCT interventions, namely facility delivery and replacement feeding, made a woman more vulnerable to passive disclosure than others. For example, both male and female study participants acknowledged the benefits of delivering in a health facility. However, health facility delivery was unaffordable for most Kibera residents and healthy women were culturally expected to deliver at home. As a result, women who delivered at a health facility were suspected of being sick, especially with HIV. Similarly, breastfeeding is the norm for mothers throughout Kenya and Kibera. Replacement feeding with formula (a recommended PMTCT strategy at the time of the study) was considered a clear indication that a woman was unable to breastfeed due to illness and was strongly associated with being HIV-positive. HIV positive women enrolled in the PMTCT program and elected to formula feed reported also breastfeeding (e.g. mixed feeding) when in the presence of others due to stigma and social pressure, especially in an effort to avoid questions from neighbors and family members. To a lesser extent, women also described how storing and taking ART medications on a daily basis was done in a covert manner to avoid questions.

*If [a mother] gives bottle feed in the open then the neighbors will start gossiping about you. But if you give bottle feed and partly breastfeed then they don't talk about you they just think you don't have much milk for the baby*.

--Woman at 1^st^ ANC Visit

### 5. Male partner support for PMTCT

A man’s support of his female partner’s enrollment and engagement in the PMTCT program was often contingent on traditional gender roles and many of the preceding themes. How well a male partner understood the opportunities to prevent vertical transmission could have an influence on his decision to support the pregnancy and remain in the relationship.

*He [male partner] told me after knowing my [HIV] status that what is the point of having a baby? He did not know that I can have a baby and it stays okay. Even now I have been thinking that I'll call him and let him know that the baby came out negative, and if he will feel like coming back then he will*.

--Woman with ≥ 1 missed appointment for PMTCT

*I think the majority of men feel that it is the unborn baby who is going to be [HIV] infected. I don't think if many of them are aware that there are other ways that they can resort to in order for the baby not to get infected. Many of them believe that the unborn baby is automatically going to be infected*.

--Man residing in Kibera

A male partner could only offer support for PMTCT interventions if he was aware they were needed to protect the health of his partner and child.

*He [male partner] knows if we follow the doctor's advice we can prevent our child from getting infected. I explained to him and also the doctor told him. He reminds me when we are supposed to come for counseling and more medication, as he also comes here for his medication*.

--Woman with ≥ 1 missed appointment for PMTCT

As presented above, fear of violence or abandonment led many women to choose against active disclosure of their HIV status to a male partner. Some male partners acknowledged these fears, but described that the absence of timely disclosure during pregnancy would feel like a betrayal and make it difficult to support female partners in the future.

*…if she gets pregnant and goes to the clinic and gets tested and found to be HIV-positive, she may get scared of disclosing, so I am kept in the dark. Now I only get to find out about the situation when the baby is born, I would get really angry, so supporting my wife would be hard*.

--Man Residing in Kibera

The overall strength of the relationship and the male’s own HIV status were considerable drivers in whether the couple remained in a relationship and openly navigated PMTCT services together. Participants described that few couples felt comfortable openly discussing matters of sex and HIV, either because they lacked the skills and knowledge to initiate such a conversation, and /or feared a violent reaction. Both men and women were suspicious of each other with regard to sexual activity and additional sexual partners. Men perceived that more women had HIV and were responsible for transmitting it to their male partners, whereas women believed it was men who were spreading HIV within Kibera.

*They all know [how HIV is spread] but they are in denial…[Men] say it the women who spread it and women know it is men who spread it*.

--Woman with at Least 3 ANC Visits

Solid relationships that were stable prior to pregnancy created a safer environment for women to actively disclose their HIV status, a process which often led to the discovery of HIV seroconcordant status and the opportunity to mutually support each other in their respective HIV care, as well as prevent vertical transmission to their infant.

*The way she came and told me, I decided to come with her here at the clinic. Initially I had refused to come, but after being talked to and tested, I started to think of our small children to bring up and decided to start on drugs*.

--Supportive Male Partner

Male partners who were aware of or suspected that they were also HIV positive, or took the steps to be tested after a female partner disclosed her HIV status, were more likely to allow her to enroll and fully participate in the PMTCT program, and be supportive of following PMTCT strategies such as medication or consistent feeding practices at home.

*I knew my status when I was pregnant …I told my husband who accepted to be tested and was found positive. He was put on medication and I continued with the Septrin and we accepted our status, so we go to the clinic together*.

--Woman with at Least 3 ANC Visits

Health clinics in Kibera were viewed as places for women and children, especially for antenatal and postnatal care. All issues related to pregnancy were considered women’s business only. It was not normal for men to seek preventive care for themselves at the health clinics; a man only went if he was acutely ill. Some men viewed their partner’s attendance at the clinic and participation in PMTCT as a threat to the control they wished to exert over the women’s health and actions. Men viewed their female partner as an extension of themselves. Thus, if community members perceived a woman as HIV positive based on her clinic attendance, this could expose the HIV status of her male partner as well.

Culturally, it is believed that issues to do with ANC and children is about women not husbands…they [men] say that the moment they're seen coming with their wives here, then the neighborhood will know that something is wrong there. They may say ‘how come she is coming with her husband’?

--Service Provider

Furthermore, men were typically busy working or searching for employment opportunities during the day and not normally involved in routine pregnancy and postnatal care. Male participation in clinic visits signaled that something was “different” about a pregnancy, creating another opportunity for passive disclosure of HIV status.

*Men have a lot to do especially in the slum settings, they need to go and look for casual jobs, so they can't go to the clinic to accompany their wives and miss a job… Some men believe that they would have to sit with their wives the whole day, so they preempt coming to avoid sitting a whole day at the clinic. It is a man's belief that going to the clinic is a woman's job, it is cultural*.

--Service Provider

Male partner support for PMTCT retention and adherence to ART could take many forms. Most actively and publicly, a man would attend PMTCT appointments at the clinic with his partner. Within the household, the man could remind her to take ART medication, remind her of clinic appointments, and support following guidance received from PMTCT service providers (e.g. condom use, consistent feeding practices).

*When I found out my status, I was sick at [District Hospital]. They gave the card to this clinic but my husband took it and hid it saying that he did not have the virus. To date he is still in denial. He has, however, accepted the fact that I'm positive and is concerned about the welfare of the child. He ensures that I take my ART, he usually reminds me*.

--Woman with at Least 3 ANC Visits

A male partner could also be considered supportive without actually doing anything overt himself. Given gender dynamics within Kibera, simply acknowledging HIV status and/or not actively prohibiting his partner from attending clinic visits and following PMTCT interventions was considered supportive male behavior. Male and female study participants who described (or hypothesized about) partner support most commonly described this passive type of permission rather than active support.

*If I already know she is positive, there is nothing I would do because to me that is help she is getting. So it does not matter if she has told me or not, it's okay for her to attend the services. I will just allow her to go because she is looking for help*.

--Man Residing in Kibera

## Discussion

We conducted an in-depth qualitative study on engagement and retention in PMTCT services in an informal settlement with high HIV prevalence in Kenya. We explored reasons why women in Kibera do not enroll and remain in care throughout the cascade of PMTCT services, especially after testing HIV-positive at their first ANC visit. The results of this qualitative study drew upon multiple unique perspectives, including those of men, service providers, and women at various stages of participating in PMTCT services. Data were triangulated across in-depth interviews and focus group discussions, as well as across multiple qualitative analysts. The results reveal that pregnant women diagnosed with HIV at their first visit to ANC choose to participate in the PMTCT program by continually assessing and reacting to perceived risks and benefits in the home, community and clinic environments (Fig 1). Qualitative data are unique to the setting and population from which they were collected and limited by self-report. The current results are specific to the Kibera context, although many of the identified themes may be applicable to other areas in Kenya or informal settlement environments elsewhere in Africa. However, because our study population resides in an urban area where services are densely clustered and PMTCT services were provided free of charge by MSF, generalizability to rural settings and clinics that are entirely government funded and operated is more limited.

### Risk analysis and management

Many distinct factors contributed to how a woman assessed her individual circumstances and overcame barriers to engage in care and follow PMTCT guidelines. Consistent with previous studies, our findings suggest that uptake of PMTCT services can be facilitated by accurate knowledge and understanding of HIV and PMTCT [8–10], positive disclosure experiences, especially with a male partner who knows his own status, and active support from both the community and male partners [24]. Our study also found that male partners had a significant influence on women’s participation in PMTCT [6, 7, 9, 10]. Factors such as being in a HIV serodiscordant relationship or having a male partner who did not know their own HIV status, as well as a lack of male support for female engagement in PMTCT services posed insurmountable barriers for some women to remain active in PMTCT care. To a lesser extent, opportunity costs, including traveling upcountry, and competing priorities for work and food acquisition [9] prevented women from attending appointments at the MSF clinic, which was the only venue for accessing PMTCT care. Other studies have identified negative attitudes from clinic staff and compromised confidentially as deterrents to PMTCT participation [6–8, 10]. Women enrolled in our study did not discuss these issues explicitly, although a loss of confidentially was implicit in circumstances of passive disclosure.

Our results have extended the issue of disclosure, by examining the distinction between active disclosure and the perceived risk of passive disclosure. In addition, our study goes beyond the identification of discrete barriers and explores the complex relationship between them. The synthesis of these five themes suggests that testing HIV-positive at first ANC triggered an ongoing assessment of vulnerability and tension between current maternal safety and the future health of their infant. For women accessing PMTCT care an HIV diagnosis can initially result in shock and fear, prompting many women to evaluate the risks involved in enrollment and retention in PMTCT care. They often received their test results within the isolated context of ANC, but almost immediately needed to process their new HIV status, digest complex clinical information, begin to follow medical guidelines, and contemplate disclosure to their male partner. They had to carefully weigh the benefits and consequences associated with active participation in the PMTCT program and develop plans on how to effectively manage the anticipated risks involved.

These risks occurred in three different, but often overlapping contexts: the home and partner relationship, the community, and the clinic environments. At home, a woman was confronted with the risks of actively disclosing, such as potential violence, perceived unfaithfulness, abandonment, loss of household income, and bringing shame to her family. If she chose not to share her HIV status with her partner, she would need to manage multiple factors to avoid passive disclosure associated with taking medication, attending clinic appointments, or replacement feeding. Competing priorities such as domestic tasks, earning money, and securing food also conflicted with attending clinic visits and following PMTCT interventions.

Within the community, a woman needed to navigate situations for passive disclosure: hiding clinic attendance and covering-up or altering infant feeding practices. By attending clinic appointments, a woman faced possible exposure as an HIV patient, a loss of confidentiality for her and her partner, and lost opportunity costs of working and providing food for her family. At the clinic, a woman was seen as a mother-to-be, and expected to prioritize the health of her unborn child. But at home and in the community, women were seen primarily as wives and girlfriends. Women had to carefully weigh the benefits and consequences associated with participation in PMTCT and develop plans on how to effectively manage anticipated risks while simultaneously maintaining their dignity and expected roles as women and mothers.

### Engagement in care throughout the PMTCT cascade

PMTCT is a long-term intervention, involving multiple steps and interventions for mother and baby, and includes lifelong adherence to medication for women who elect to initiate antiretroviral therapy. Our findings illustrate that uptake of PMTCT services can manifest in a variety of ways, and engaging in PMTCT care is best viewed as a continuum rather than being categorized as a discrete yes or no.

Some women may have been unaware of the benefits of PMTCT or were too fearful of HIV stigma, thereby avoiding ANC HIV testing and PMTCT entirely because they concluded from their initial risk assessment that even the possibility of an HIV diagnosis was too much for them to navigate. This may explain why some women were lost to follow-up from the outset; they tested positive for HIV at their 1^st^ ANC but never came back to the clinic and could not be traced by a social worker. Importantly, our results did not include the perspective directly from patients who were initially lost to follow-up because we were unable to contact these women to reengage in care or be recruited for our study. These women may have been unprepared for an HIV diagnosis, did not feel that they could disclose to their partner, or did not have coping mechanisms to manage the anticipated risks associated with PMTCT. Alternatively, clinic-level factors or migration may have led them to seek care, including PMTCT services, at a different health facility. A pooled estimate from a recent meta-analysis found that when HIV patients initially categorized as LTFU were intensively traced, 18% were receiving care at a different facility [25]. These “silent transfers” can contribute to an overestimate of a program’s true LTFU rate. Active patient tracing is resource-intensive, but serves to more accurately categorize patients’ status in care, as well reinforce key PMTCT counselling messages in real-time and encourage continued engagement in care. The MSF Kibera clinics have since implemented an active patient tracing system [26].

Other women missed appointments occasionally and complied with some, but not all, aspects of PMTCT guidelines. They may have intended to fully engage in PMTCT services and interventions, but along the way they encountered barriers and had to make difficult choices. Disclosure to a male partner was a strong determining factor in female clinic attendance and participating in PMTCT. If a woman did not feel able to disclose she may still have participated in PMTCT “undercover” without explaining her HIV diagnosis. However, this condition would make it more challenging to fully engage in and adhere to PMTCT services and interventions.

Women who were able to attend clinic visits and fully follow the PMTCT guidelines and counselling seemed to have one or more facilitating factors present in their daily life, such as strong knowledge of PMTCT, a supportive partner, and/or reasonable communication with their partner. These facilitators, combined with their understanding of the benefits of PMTCT, helped these women overcome any perceived risks and develop a strategy to remain active in the PMTCT program for the duration of their pregnancy.

### Implementation of PMTCT services

PMTCT programs have traditionally focused on the clinic environment. However, the results of this study suggest that most factors influencing overall adherence to PMTCT take place outside the clinics, within the home and community environments. Many women were confronting HIV for the first time at ANC and needed to adapt to their new diagnosis quickly. Our findings lead to suggestions for changes in the overall approach to service delivery for PMTCT that are applicable to Kibera as well as other similar resource-limited settings.

More effective delivery of PMTCT services requires a community-based public health approach as well as patient-centered, efficacious clinical interventions and efficient clinic management. Community understanding of, support for, and involvement in PMTCT services are needed to reduce HIV-related stigma [7, 9, 10, 24, 27]. Education on the benefits of HIV testing, earlier initiation of ANC, and PMTCT need to be delivered to the community in advance of pregnancy and widely to men, women, and adolescents. Increasingly, male involvement in PMTCT services has been identified as a promising practice to improve uptake of and retention of these interventions [6, 7, 10, 28], and our study supports this finding. Couples testing can facilitate disclosure, improve communication, and help navigate issues of HIV serodiscordance and PMTCT. One innovative strategy to increase HIV testing of male partners is to offer home-based couples counselling and testing. Offering testing services in the home addresses barriers that prevent men from joining their pregnant partners at the health clinic and a trained counselor can facilitate mutual disclosure of HIV status [29]. Provision of peer mentoring, highlighting female and male PMTCT success stories and role models, is another promising practice to provide community-based social support and complement clinic-based adherence counselling [8–10, 27]. Extending clinic hours and reducing wait times, to accommodate working men and women and improved integration of HIV/PMTCT services into routine primary and antenatal care services may reduce the stigma of seeking care [28, 30], and make clinics more inviting to men. Financial insecurity had a significant impact on initiating and maintaining engagement with health care services, including women’s financial dependence on male partners and the need for both men and women to prioritize income generating activities over clinic visits. These results highlight the role that community partners, such as social service and microfinance agencies, can play in creating a community network that is supportive of HIV prevention.

It is important to consider these results within the context of the WHO’s current recommendations for prevention of vertical transmission of HIV. Starting in 2013, WHO PMTCT guidelines recommend that newly diagnosed HIV-positive pregnant women initiate lifelong ART immediately, regardless of CD4 count (Option B+). This approach is intended to streamline clinic operations and has the potential to be cost-effective [31, 32]. However, although initiation of ART has increased substantially in settings that have adopted this new strategy, the overall effectiveness of PMTCT Option B+ is still hampered by low adherence to ART and high attrition of mothers and infants. In Malawi, where Option B+ was first implemented, ART initiation among HIV-positive pregnant women increased by 11% in the first year of implementation, but LTFU by six months was 58% at some clinics and highest within the first three months post ART initiation [33]. While the core recommendation of Option B+, immediate initiation of lifelong ART and exclusive breastfeeding, are more straightforward than the earlier guidelines being implemented at the time of the current study, a simplified ART protocol alone is not sufficient for PMTCT programs to succeed in resource-limited settings. In fact, by initiating lifelong treatment, including the entire breastfeeding period, current PMTCT guidelines increase the imperative to continue addressing the barriers identified here to successfully engage and retain women in care long term [6, 10]. Rollout of ART in Sub-Saharan Africa has increased substantially in recent years. Increased access to ART, simplified drug regimens, and restored health have motivated thousands of HIV infected individuals to initiate and maintain ART [34], however, HIV-related stigma continues to undermine ART adherence in some populations and settings [35].

The results of the current study underscore the importance of individualized counselling and community support throughout the PMTCT cascade, and this must be implemented in tandem with WHO clinical guidelines. After a pregnant woman has been diagnosed with HIV, a patient-centered approach to PMTCT should be immediately implemented that is customized to her unique circumstances. Effective counselling has been found to be one of the most influential factors for women’s adherence, especially when disclosure to a male partner is facilitated [8]. Our results support the recommendation that counseling should include a discussion of a woman’s unique perceptions of risk, especially if and how to safely disclose to her male partner. ANC is a critical, but often brief, opportunity to engage women and their families in HIV treatment and prevention. An upfront investment in counseling and decision making at the time a woman enrolls in a PMTCT program may facilitate treatment adherence throughout and after pregnancy.

## Conclusion

The results of this study illustrate the complexity of living with HIV in the setting of an informal settlement like Kibera, and the unique challenges that HIV-positive pregnant women face. This study goes beyond describing discrete barriers to participating in PMTCT services, and explains a dynamic analysis of actual and perceived risks in the home, community, and clinic that begins when a woman is diagnosed with HIV at her first ANC visit. This risk analysis is unique to each woman, and can change throughout the course of her pregnancy, informing her engagement in PMTCT services. The risk analysis is strongly influenced by a woman’s male partner, even if he does not yet know her HIV status. Our study reveals that the day-to-day realities that pregnant women living with HIV in Kibera must negotiate, especially dynamics with male partners, make it challenging to follow PMTCT counselling and interventions provided at the clinic. Engaging the community to actively address social factors outside of the clinic environment, ongoing patient-tailored counseling for HIV-positive pregnant women, and increasing male involvement are key to the success of PMTCT programs in Kibera and similar settings.
